# Supplementary material for: Gram-Scale Synthesis of Graphitic Carbon Nitride Quantum Dots with Ultraviolet Photoluminescence for Fe3+ Ion Detection
Source: Nanomaterials (Basel). 2022 Aug 16;12(16):2804. doi: 10.3390/nano12162804 (PMC9413325; doi:10.3390/nano12162804)
Supplement: Supplementary file 1 [file nanomaterials-12-02804-s001.zip › nanomaterials-1833844-SI.pdf]

# Supporting Information for

# Gram-scale synthesis of graphitic carbon nitride quantum dots with ultraviolet photoluminescence for $Fe^{3+}$ ion detection

**Xuemei Lu, Haijun Qin, Jiuzhang Cai, Yuhang Cui, Lixin Liao, Fengzhen Lv, Changming Zhu, Liguang Wang, Jun Liu, Lizhen Long, Wenjie Kong and Fuchi Liu***

College of Physics and Technology & Guangxi Key Laboratory of Nuclear Physics and Technology, Guangxi Normal University, Guilin 541004, China

* Correspondence: liufuchi@gxnu.edu.cn

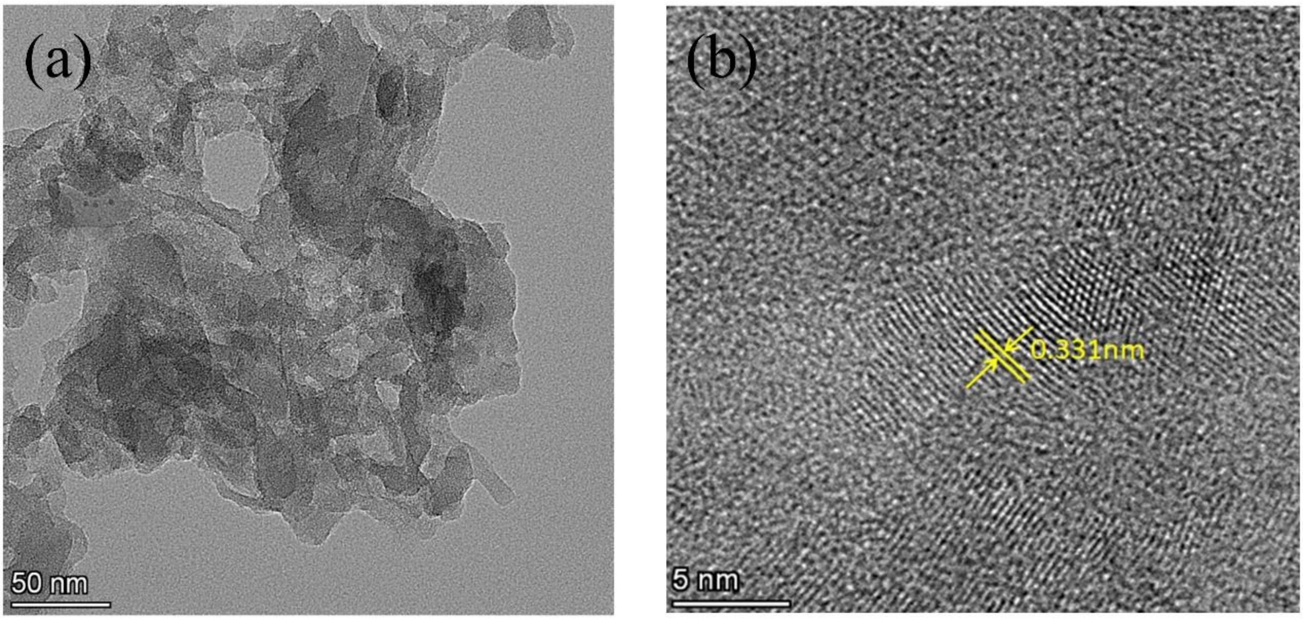


**Figure S1.** TEM image(a) and HR-TEM image(b) of g-$C_3N_4$.

Figs. S1(a) and S1(b) are TEM and HR-TEM images of g-$C_3N_4$, the original layered structure of g-$C_3N_4$ is obvious, and the clear lattice spacing of g-$C_3N_4$ obtained by HR-TEM was approximately 0.331 nm.

**Table S1.** The relative ratios of C-C/C-N, C-O-C, N-C=N, C=O, O-C=O, and C-$CO_3$ of each sample in C 1s spectral analysis.

| Samples/ Chemical bonds | g-$C_3N_4$ | | g-$C_3N_4$QDs | |
|---|---|---|---|---|
| | Area | % | Area | % |
| C-C/C-N | 10726.23 | 6.53 | 58105.75 | 26.16 |
| C-O-C | 13785.41 | 8.41 | 7114.014 | 3.20 |
| N-C=N | 111992.8 | 68.36 | 17477.94 | 7.87 |
| C=O | 18208.48 | 11.12 | 20954.85 | 9.43 |
| O-C=O | / | / | 118478.4 | 53.34 |
| C-$CO_3$ | 9110.59 | 5.58 | / | / |

**Table S2.** The relative ratios of C-N=C, N-(C)3, C-N-H and π-π* in N 1s spectral analysis.

| Samples/ | $g-C_3N_4$ | | $g-C_3N_4QDs$ | |
|---|---|---|---|---|
| Chemical bonds | Area | % | Area | % |
| C-N=C | 238982 | 68.64 | 70847.24 | 25.34 |
| N-(C)3 | 71858.33 | 20.64 | 89414 | 31.98 |
| C-N-H | 14840.59 | 4.26 | 116874.7 | 41.80 |
| π-π* | 22477.61 | 6.46 | 2469.408 | 0.88 |

**Table S3.** The relative ratios of C-O-C, C=O and COOH in each sample in O 1s spectral analysis.

| Samples/ | $g-C_3N_4$ | | $g-C_3N_4QDs$ | |
|---|---|---|---|---|
| Chemical bonds | Area | % | Area | % |
| C-O-C | 13677.05 | 80.17 | 123993.8 | 36.26 |
| C=O | 3383.893 | 19.83 | 147737.6 | 43.21 |
| COOH | / | / | 70195.02 | 20.53 |

As shown in Table S1 and S3, the oxygen element in $g-C_3N_4$ exists in the form of oxygen-containing functional groups (C-O-C, C=O) without COOH, and COOH is mainly formed during oxidation and reflux. As shown in Table S2, the content of C-N=C in $g-C_3N_4QDs$ is lower than $g-C_3N_4$, indicating that the triazine unit of $g-C_3N_4$ is damaged and the oxygen element is introduced in the process of oxidation and reflux.

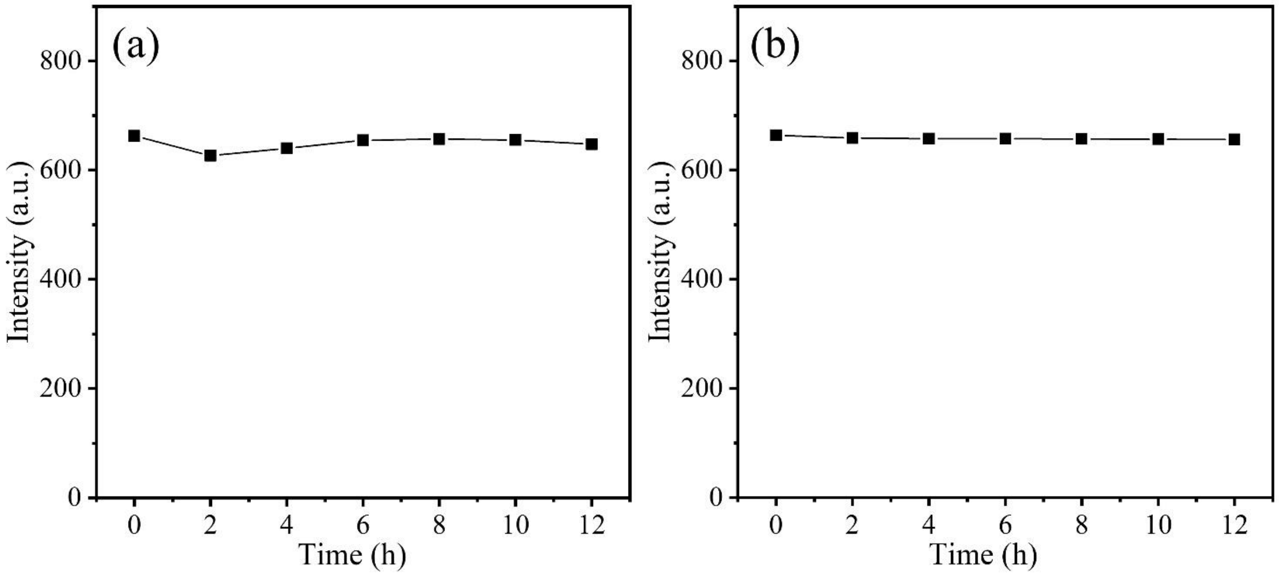


**Figure S2.** Fluorescence of $g-C_3N_4QDs$ under irradiation by (a) visible light, and (b) 365 nm within 12 h.

It can be seen from the figure that the fluorescence intensity of $g-C_3N_4QDs$ is almost stable under the continuous irradiation of visible light /365 nm for 12 h.

**Table. S4.** 15 groups of blank g-$C_3N_4$QDs fluorescent probes.

| Test | PL (Intensity) | Test | PL (Intensity) | Test | PL (Intensity) |
|---|---|---|---|---|---|
| 1 | 408.467 | 6 | 408.375 | 11 | 408.369 |
| 2 | 409.365 | 7 | 409.586 | 12 | 409.861 |
| 3 | 408.695 | 8 | 407.251 | 13 | 408.894 |
| 4 | 408.335 | 9 | 409.029 | 14 | 408.316 |
| 5 | 409.025 | 10 | 407.917 | 15 | 409.157 |

As shown in Table. S4, we measured 15 groups of blank g-$C_3N_4$QDs fluorescent probes and their standard deviation was calculated to be 1.5397.
